# Hybrid Bioprinting of Chondrogenically Induced Human Mesenchymal Stem Cell Spheroids

**DOI:** 10.3389/fbioe.2020.00484

**Published:** 2020-05-25

**Authors:** Lise De Moor, Sélina Fernandez, Chris Vercruysse, Liesbeth Tytgat, Mahtab Asadian, Nathalie De Geyter, Sandra Van Vlierberghe, Peter Dubruel, Heidi Declercq

**Affiliations:** ^1^Tissue Engineering Group, Department of Human Structure and Repair, Faculty of Medicine and Health Sciences, Ghent University, Ghent, Belgium; ^2^Polymer Chemistry and Biomaterials Research Group, Department of Organic and Macromolecular Chemistry, Faculty of Sciences, Centre of Macromolecular Chemistry, Ghent University, Ghent, Belgium; ^3^Research Unit Plasma Technology, Department of Applied Physics, Faculty of Engineering and Architecture, Ghent University, Ghent, Belgium; ^4^Tissue Engineering Lab, Department of Development and Regeneration, Faculty of Medicine, KU Leuven Kulak, Kortrijk, Belgium

**Keywords:** bioprinting, spheroids, chondrogenesis, differentiation, stem cell, fusion, self-assembly

## Abstract

To date, the treatment of articular cartilage lesions remains challenging. A promising strategy for the development of new regenerative therapies is hybrid bioprinting, combining the principles of developmental biology, biomaterial science, and 3D bioprinting. In this approach, scaffold-free cartilage microtissues with small diameters are used as building blocks, combined with a photo-crosslinkable hydrogel and subsequently bioprinted. Spheroids of human bone marrow-derived mesenchymal stem cells (hBM-MSC) are created using a high-throughput microwell system and chondrogenic differentiation is induced during 42 days by applying chondrogenic culture medium and low oxygen tension (5%). Stable and homogeneous cartilage spheroids with a mean diameter of 116 ± 2.80 μm, which is compatible with bioprinting, were created after 14 days of culture and a glycosaminoglycans (GAG)- and collagen II-positive extracellular matrix (ECM) was observed. Spheroids were able to assemble at random into a macrotissue, driven by developmental biology tissue fusion processes, and after 72 h of culture, a compact macrotissue was formed. In a directed assembly approach, spheroids were assembled with high spatial control using the bio-ink based extrusion bioprinting approach. Therefore, 14-day spheroids were combined with a photo-crosslinkable methacrylamide-modified gelatin (gelMA) as viscous printing medium to ensure shape fidelity of the printed construct. The photo-initiators Irgacure 2959 and Li-TPO-L were evaluated by assessing their effect on bio-ink properties and the chondrogenic phenotype. The encapsulation in gelMA resulted in further chondrogenic maturation observed by an increased production of GAG and a reduction of collagen I. Moreover, the use of Li-TPO-L lead to constructs with lower stiffness which induced a decrease of collagen I and an increase in GAG and collagen II production. After 3D bioprinting, spheroids remained viable and the cartilage phenotype was maintained. Our findings demonstrate that hBM-MSC spheroids are able to differentiate into cartilage microtissues and display a geometry compatible with 3D bioprinting. Furthermore, for hybrid bioprinting of these spheroids, gelMA is a promising material as it exhibits favorable properties in terms of printability and it supports the viability and chondrogenic phenotype of hBM-MSC microtissues. Moreover, it was shown that a lower hydrogel stiffness enhances further chondrogenic maturation after bioprinting.

## Introduction

Articular cartilage, the connective tissue lining the articular surface of bones within diarthrodial joints, ensures load support, load transmission, and joint lubrication. It is characterized by a limited intrinsic healing and repair capacity because of its avascular and aneural nature. Therefore, articular cartilage lesions are prone to progress to osteoarthritis and patients are liable to suffer from joint instability in the long term (Fox et al., 2009, 2012).

Nowadays, articular cartilage defects are already being treated with cell-based regenerative therapies, such as autologous chondrocyte implantation (ACI). This two-step surgical procedure includes the isolation of autologous articular chondrocytes from a non-weight bearing region, the *in vitro* expansion and the reinjection into the defect site (Davies and Kuiper, 2019). Another commonly used approach is Matrix-assisted ACI (MACI) which is an *ex vivo* engineered hybrid construct, where isolated autologous chondrocytes are seeded onto a biomaterial, a bovine-derived type I/III collagen scaffold, before implantation in the defect (Foldager et al., 2012; Basad et al., 2015). However, the harvesting procedure to obtain chondrocytes can induce donor-site morbidity and the 2D expansion of articular chondrocytes is characterized by long *in vitro* culture periods and chondrocyte dedifferentiation to a more fibroblast-like phenotype featuring a decrease in collagen II, aggrecan, and glycosaminoglycans (GAG) (Caron et al., 2012; De Moor et al., 2019). This results in the generation of repair tissue which is biochemically and hence biomechanically inferior compared to the native cartilage (Knutsen et al., 2007). Therefore, new biofabrication strategies are being explored in the quest for novel regenerative therapies.

For the creation of cartilage constructs, a modular tissue engineering concept is emerging. This approach is inspired by developmental biology where complex tissues are comprised of repeating functional units (Nichol and Khademhosseini, 2009). Instead of starting from single cells, smaller tissue units such as cell sheets or cellular spheroids are used as building blocks to create larger tissues by self-assembly and fusion. The 3D spatial arrangement of cells into spheroids mimics the natural environment by creating cell–cell contacts and cell–extracellular matrix (ECM) interactions, enhancing cell differentiation (Mironov et al., 2009; Schon et al., 2017). Injection of spheroids can be used to treat cartilage defects instead of injecting a single cell suspension, whose dedifferentiation leads to inferior fibrous tissue. Spheroid maturation already starts *in vitro*, establishing a tissue specific ECM provoked by their high cellularity and cell density (Huang et al., 2016; De Moor et al., 2019). An example used in clinical trials are chondrospheres, 500–800 μm spheroids generated from autologous chondrocytes. The chondrospheres naturally adhere to the cartilage defect after injection and assemble and fuse at random (Anderer and Libera, 2002; Becher et al., 2017).

As an alternative to injecting or assembling spheroids at random, 3D printing technologies can be used to assemble the cellular building blocks in a directed manner with high spatial control in a complex predesigned configuration. In bio-ink based extrusion bioprinting, biomaterials can be used as viscous carriers for the deposition of cells or spheroids, to restrain them and to ensure shape fidelity of the printed construct (Moldovan et al., 2018). Moreover, hydrogels are widely used in cell-based cartilage regeneration as a pro-chondrogenic environment as they can mimic the biological and physical properties of the native ECM (Vega et al., 2017). Next to alginate or agarose hydrogels, commonly used for chondrocyte redifferentiation, gelatin-based hydrogels are an optimal candidate to use as an ink because of their low-cost, high water content, cell-interactive properties and resemblance with the natural ECM as a derivative from collagen (Cigan et al., 2016; Ewa-Choy et al., 2017; Pahoff et al., 2019). Methacrylamide-modified gelatin (gelMA), also frequently referred to as gelatin methacryloyl in the literature, is a modified photo-crosslinkable form of the natural polymer gelatin combining stability (after crosslinking) at 37°C and allowing interaction, adhesion and migration of cells by the presence of the integrin binding Arg-Gly-Asp (RGD) sequence. GelMA solutions are interesting for extrusion bioprinting as thermally initiated (reversible) gelation can create a viscous gel-like solution, ideal for extrusion, by applying a printing temperature below 30°C (Van Den Bulcke et al., 2000; Pepelanova et al., 2018). GelMA can be chemically crosslinked by a UV-induced chain growth polymerization mechanism in the presence of a photo-initiator (PI), which transfers the electromagnetic energy of the UV-light into chemical energy by generating radicals and initiating the polymerization (Tytgat et al., 2018). Hydrogel properties can be tailored by varying polymer concentration, crosslinking times or by using different PIs (Rouillard et al., 2011; Billiet et al., 2014; Loessner et al., 2016; Pahoff et al., 2019). Irgacure 2959 is a commonly used PI but its water solubility is very limited, therefore there is a shift to PIs with higher water solubility such as Li-TPO-L (Markovic et al., 2015; Tytgat et al., 2018). It has been demonstrated that gelMA supports cartilage-like matrix production when a suspension of single chondrocytes or mesenchymal stem cells (MSC) were embedded (Hu et al., 2009; Schuurman et al., 2013; Mouser et al., 2018; Pahoff et al., 2019). Recently, there has been an increased interest in the use of adult MSC in cartilage regeneration because of their multipotency and ability to differentiate into chondrogenic lineages after expansion *in vitro* and intra-articular injections of MSC have been performed to reduce osteoarthritic pain or induce cartilage repair (Jo et al., 2014; Shin et al., 2018). The use of MSC would also eliminate the need for harvesting surgery within the damaged knee-joint or donor site. Spheroid formation of stem cells resembles the aggregation and condensation processes of mesenchymal progenitor cells during embryonic cartilage development (Ghosh et al., 2009). Next to culturing MSC in 3D, differentiation into the chondrogenic lineage can be induced by applying a chondrogenic culture medium containing growth factors such as transforming growth factor-β (TGF-β) and/or bone morphogenetic protein (BMP) (Ude et al., 2017). Further mimicking of the natural environment can be done by culturing in a low oxygen environment as hypoxia results in an increase of cartilage ECM molecules such as collagen II and aggrecan (Tan et al., 2011; Berneel et al., 2016).

**FIGURE 1:**
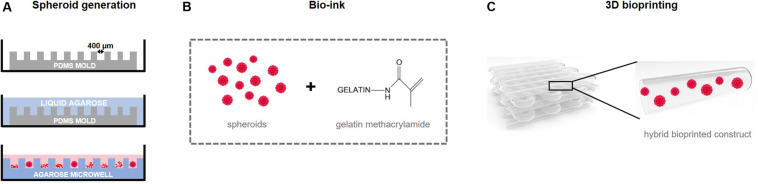
Experimental study design. **(A)** Printable micro building blocks (spheroids) for the creation of a larger construct are generated in high-throughput using non-adhesive microwells. Agarose microwells are created by using a PDMS mold as a negative replica. After seeding, cells lower into the pores of the microwell and self-assemble into 3D cellular spheroids. **(B)** Spheroids are combined with a hydrogel precursor solution, a methacrylamide-modified gelatin for **(C)** 3D bio-ink based bioprinting of a construct using extrusion.

For hyaline cartilage tissue engineering, mainly large diameter spheroids (>500 μm) are created using articular chondrocytes (Anderer and Libera, 2002; Armoiry et al., 2018). Though for bioprinting, small diameter spheroids with uniform shapes and sizes are necessary for compatibility with the print needle. In our previous study, we described the biofabrication of printable high quality cartilage microtissues starting from porcine chondrocytes (De Moor et al., 2019). However, to enhance clinical translation, we describe the high-throughput creation of small diameter human cartilage spheroids (<200 μm) by chondrogenic differentiation of human bone marrow-derived mesenchymal stem cells (hBM-MSC), applying chondrogenic culture medium and low oxygen tension (5%). To generate the spheroids, a low-cost non-adhesive microwell system is used (Figure 1). Moreover, the fusion capacity of the cartilage microtissues in suspension and within a hydrogel is investigated. We investigated if gelMA is a suitable conductive microenvironment for spheroid differentiation by extensive screening of ECM by histology. In the final phase of the study, the processing potential of the bio-ink, consisting of cartilage microtissues and gelMA, and the impact of the extrusion-based bioprinting on viability and cartilage phenotype were assessed (Figure 1).

## Materials and Methods

### Cell Culture

Human Bone Marrow-derived Mesenchymal Stem Cells were purchased from PromoCell GmbH (C-12974, male donor age 65). Cells were cultured in MSC Growth Medium 2 (C-28009, PromoCell GmbH) supplemented with 50 U/ml penicillin and 50 μg/ml streptomycin (Life technologies) and maintained at 37°C in a humidified 5% CO_2_-containing atmosphere. Cells were subcultured at 80% confluency. Passage 3–5 cells were used to generate spheroids in this study. Passage 3 cells were applied in the fusion experiments and passage 4 and 5 cells were used in all other experiments.

### Fabrication of Non-adhesive Microwells

Spheroids are generated using a high-throughput non-adhesive agarose microwell system, as previously described (Gevaert et al., 2014; Berneel et al., 2016; Roosens et al., 2017; De Moor et al., 2018, 2019). In brief, polydimethylsiloxane (PDMS) molds (NaMiFab, Ghent University) with a diameter of 18 mm and a height of 3 mm, containing 1585 micropores of 400 μm diameter each, are used as a negative replica to create microwells (Figure 1A). A 4.5 w/v% Ultrapure agarose solution (Life technologies) dissolved in sterile phosphate buffered saline (PBS) was heated and poured on top of the PDMS mold. Once the agarose solidified, the agarose microwell was separated from the mold and placed in a 12-well plate.

### Generation of 3D Microtissues

Cells were harvested and 500 μl of cell suspension, containing 5.0 × 10^5^ cells, was seeded onto the microwell, resulting in approximately 315 cells per pore. One hour after seeding, cells lowered into the pores by gravitational force. To induce chondrogenesis, spheroids were cultured in a serum-free chondrogenic culture medium comprised of Dulbecco’s Modified Eagle Medium/Nutrient Mixture F-12 (DMEM/F12, Life Technologies), 1 mM sodium pyruvate (Life technologies), 0.5% (v/v) ITS (Sigma-Aldrich), 10 U/mL penicillin, 10 μg/mL streptomycin, 100 μM dexamethasone (Sigma-Aldrich), 200 μM L-ascorbic acid-2-phosphate (Sigma-Aldrich), and 10 ng/mL TGF-β1 (Peprotech), in a low oxygen tension (5% O_2_) incubator at 37°C. Spheroids were cultured up to 42 days and culture medium was refreshed after the first 24 h of culture and afterward every 2 days. Aggregation of the cells was evaluated microscopically (Olympus IX81) and spheroids were harvested after 7, 14, 21, 28, 35, and 42 days of culture. Spheroid morphology was analyzed with the Xcellence image software (Olympus). Spheroid diameter, area (A) and perimeter (p) were measured and circularity was calculated using the formula *f*_*circularity*_ = (4πA)/p^2^. For the evaluation of diameter and circularity, 18 spheroids (*n* = 3), were assessed.

### At Random Assembly of Spheroids: Fusion Assays

As spheroids were used as building blocks to create larger tissues, their capacity to fuse at random was tested by using two methods.

#### Fusion of Doublets

**FIGURE 2:**
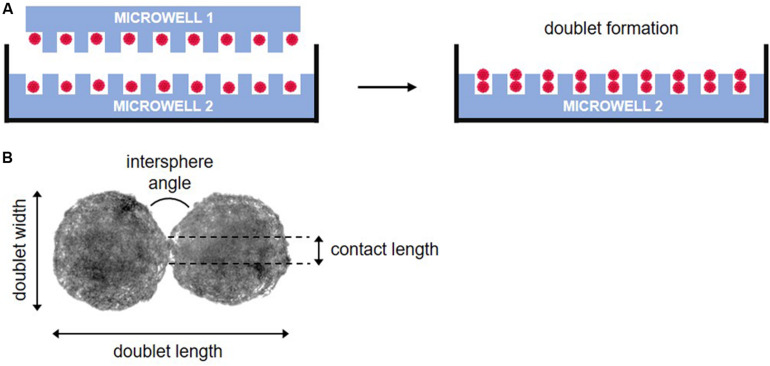
Schematic representation of doublet fusion assay. **(A)** Spheroids are pairwise placed by inverting a donor microwell (microwell 1) on top of a recipient microwell (microwell 2) and after centrifugation, the donor microwell was removed, leaving spheroid doublets in microwell 2. **(B)** Morphological parameters measured during fusion.

Spheroid doublets were formed based on the method of Susienka et al. (2016). Medium was carefully removed from two microwells containing immature 7-days-cultured spheroids. The recipient chip (microwell 2) was then transferred into a new 12-well plate. The walls of the donor chip (microwell 1) were removed using a punching device and the remaining central portion was gently inverted onto the recipient chip. After centrifugation, the donor chip was removed leaving the recipient chip with numerous spheroid doublets and 2 ml of chondrogenic medium was gradually added (Figure 2A). Images of 14 doublets were analyzed after 0, 2, 6, 8, 10, 24, 48, 72, 96, and 168 h of culture. Intersphere angle, doublet length, doublet width, and contact length (indicated in Figure 2B) of the fused doublets were measured in ImageJ.

#### Fusion of Multiple Spheroids

To assess the capacity of multiple spheroids to fuse into a macrotissue, 7-day-old spheroids were fused in an agarose coated well. A 2 w/v% agarose solution was prepared and a coating was applied in a 96-well plate (Greiner) by pipetting 300 μl per well and subsequently removing 270 μl. After 7 days of culture, spheroids of one microwell were harvested and resuspended in 160 μl chondrogenic medium. Per agarose coated well, 20 μl of the spheroid suspension was seeded, resulting in 200 spheroids per well. After seeding, 200 μl of chondrogenic medium was carefully added. Follow-up of the at random fusion was carried out at several time points: 0, 2, 6, 8, 24, 48, 72, 96, and 168 h.

### Biopolymer and Photo-Initiator Preparation

Methacrylamide-modified gelatin was provided by the Polymer Chemistry and Biomaterials Research group (Ghent University) and was prepared by functionalization of the primary amines of the (hydroxy)lysine and ornithine side groups present in bovine type B gelatin (Rousselot) with methacrylic anhydride (Sigma-Aldrich) (Van Den Bulcke et al., 2000; Van Hoorick et al., 2017; Tytgat et al., 2019). Briefly, 100 g gelatin (0.000385 mol amines/g) was dissolved in 1 L phosphate buffer (pH 7.8) at 40°C. One equivalent of methacrylic anhydride (0.0385 mol, 5.7 ml) was added dropwise while stirring for 1 h. The mixture was dialyzed against distilled water using dialysis membranes for 24 h. The degree of substitution of purified gelatin methacrylamide was 78%, as determined by ^1^H-NMR spectroscopy (Bruker AVANCE II 500 MHz). Freeze-dried gelMA was sterilized by a cold ethylene oxide treatment (AZ Sint-Jan, Bruges, Belgium) before use. 10 w/v% solutions of gelMA, dissolved in sterile autoclaved PBS while stirring at 37°C, were used. To initiate photo-crosslinking, Irgacure 2959 (1-[4-(2-hydroxyethoxy)-phenyl]-2-hydroxy-2-methyl-1-propane-1-one) (BASF) or lithium phenyl-2,4,6-trimethylbenzoylphosphinate (Li-TPO-L or LAP) (kindly provided by the Polymer Chemistry and Biomaterials Research group, Ghent University, synthesized as previously reported by Markovic et al. (2015), dissolved in PBS, were used as PI. 2 mol%, relative to the amount of methacrylamides present was added to the gelMA solution. Before adding, stock solutions of 0.8 w/v% of Irgacure or Li-TPO-L were sterilized using a 0.22 μm pore filter (Millipore).

### Hydrogel Characterization

To evaluate the effect of different PIs on the hydrogel properties, gel fraction, swelling ratio and Young’s moduli of cell-free gelMA/Irgacure 2959 or gelMA/Li-TPO-L hydrogel samples were determined. Hydrogel disks (diameter = 10 mm, height = 2 mm) were prepared by pipetting 800 μl of a 10 w/v% gelMA solution, containing 2 mol% Irgacure 2959 or Li-TPO-L, relative to the amount of methacrylamides present in gelMA, in a 6-well plate. After 10 min of physical gelation at room temperature (RT), gels with Irgacure 2959 as PI were photo-crosslinked for 20 min with a UV-A broad spectrum light (365 nm, 4 mW/cm^2^, UVP Inc.), gels with Li-TPO-L were crosslinked using a UV-LED incorporated in the RegenHu bioprinter for 60 s (365 nm, 200 mW/cm^2^). Disks of 10 mm diameter were punched out using a punching device.

#### Gel Fraction and Swelling Ratio

Gel fraction and swelling ratio of the gels was determined. Samples were dried in a desiccator for 2 weeks and weighed (*W_*d*__0_*), followed by swelling overnight in PBS at 37°C and weighed again (*W*_*s*_). Subsequently, the samples were dried and weighed again (*W_*d*__1_*). The gel fraction and swelling ratio were defined as:

$$Gelfraction\left(\%\right)=\frac{W_{d\,1}}{W_{d\,0}}\times 100$$

$$Swellingratio\left(\%\right)=\frac{W_{s}-W_{d\,0}}{W_{d\,0}}\times 100$$

#### Compression Test

After crosslinking, hydrogel disks were compressed using a universal testing machine (LRXplus, Lloyd Instruments) equipped with a 100 N load cell, at a rate of 5 mm/min. Hydrogel disks were compressed at RT over a distance of 1 mm and Young’s moduli (kPa) were calculated. All measurements were prepared in fivefold.

### Encapsulation of Spheroids in gelMA

Spheroids were combined with gelMA as a viscous printing medium for bio-ink based bioprinting by extrusion (Figure 1B). To examine the effect of hydrogel properties and PI type on spheroid morphology, viability and phenotype, hydrogel encapsulation experiments without printing were performed. Spheroids were encapsulated in 10 w/v% gelMA containing either Irgacure 2959 or Li-TPO-L as PI. Spheroids were collected from the microwells after 14 days of culture by vigorously resuspending the culture medium. After centrifugation, supernatant was removed and the gelMA/PI solution was added to the spheroid pellet. Hydrogel disks of 250 μl were prepared by pipetting spheroid/gelMA/PI solution in a 48-well plate. Per 250 μl gel, spheroids of 3 microwells were encapsulated resulting in ±4755 spheroids/gel. After physical crosslinking for 10 min at RT, gelMA/Irgacure 2959 gels and gelMA/Li-TPO-L gels were photo-crosslinked as described above (see section “Hydrogel Characterization”). Gels were transferred to a 12-well plate and chondrogenic medium was added. Medium was exchanged after 24 h and then every other 2 days, encapsulated spheroids were cultured for 14 days.

### Directed Assembly of Spheroids: 3D Extrusion-Based Bioprinting

The processing potential of the bio-ink, gelMA/Li-TPO-L containing 14-day-old spheroids, was evaluated using extrusion-based 3D bioprinting, to create controlled formation of a larger construct (Figure 1C). Spheroids from 8 microwells (±12680 spheroids) were collected by vigorously resuspending the culture medium. After centrifugation, supernatant was removed and 1.0 ml of a 10 w/v% gelMA/Li-TPO-L solution was added to the pellet of spheroids. As a control, constructs containing single cells instead of spheroids were also printed. 4 × 10^6^ hBM-MSC were collected and 1.0 ml of a 10 w/v% gelMA/Li-TPO-L solution was added to the cell pellet. After homogenization, the bio-ink solution was transferred into a cartridge and placed into the cartridge heater at 25°C for 30 min. Scaffolds were produced using the 3D Discovery Instrument (RegenHU), operated by human machine interface (HMI) and BioCAD software, equipped with a pneumatic dispensing printhead. A polyethylene conical needle with an inner diameter of 0.41 mm was used to print the scaffold by extrusion at feed rate of 5 mm/s. The printed scaffolds (1.3×1.3 cm) contained four layers of bio-ink (two horizontal, two vertical), printed with a theoretical strut thickness of 0.328 mm (80% of the needle’s inner diameter). A pressure of approximately 0.035 MPa was applied and manually adjusted during the printing process of the constructs. After printing, scaffolds were physically crosslinked at RT for 10 min and photopolymerization of the scaffolds was induced by the UV-LED incorporated in the RegenHu bioprinter for 60 s (365 nm, 200 mW/cm^2^). All printing parameters are described in Table 1.

**TABLE 1:** Printing parameters.

**Parameter**	**Value**
Cartridge temperature	25°C
Extrusion pressure	0.035 MPa
Ambient temperature	21°C
Feed rate	5 mm/s
Needle diameter	0.41 mm
Theoretical strut thickness	0.328 mm
Layers	4
Spheroid concentration	±12680/ml
Physical crosslinking	10 min at 4°C
UV irradiation time	60 s

### Scanning Electron Microscopy

Bioprinted scaffolds containing cartilage microtissues were washed two times with PBS and fixed in 2% glutaraldehyde in 0.1 M cacodylatebuffer (pH 7.2) for 3 h at RT. Next, scaffolds were washed three times in 0.1 M cacodylatebuffer, dehydrated in graded alcohol concentrations (30 min of 50%, 70%, 85%, and 95%) and completely dried by using hexamethyldisilazane (Sigma-Aldrich). Once dried, scaffolds were first coated with a thin layer of gold making use of a sputter coater (JFC-1300 auto fine coater, JEOL) to avoid charge accumulation. Scanning electron microscopy (SEM) (JSM-6010 PLUS/LV; JEOL) images were used to examine the morphology of the scaffolds and spheroids, and were obtained with an accelerating voltage of 7 kV at a working distance of 11 mm.

### Live/Dead Viability Assay

To determine cell viability, spheroids, hydrogel samples or bioprinted scaffolds were harvested, two times washed with PBS and incubated with calcein-AM (2 μg/ml, Anaspec) and propidium iodide (2 μg/ml, Sigma). After 10 min of incubation, spheroid viability was evaluated using an inverted fluorescence microscope (Olympus IX81) equipped with Xcellence software (Olympus).

### (Immuno)Histochemical Evaluation of Proliferation and Extracellular Matrix Components

Spheroids of 2 microwells were collected and pooled for histological analysis. Hydrogels and bioprinted scaffolds were rinsed with PBS 2 times prior to fixation. All samples were fixed overnight at 4°C with 4% paraformaldehyde, dehydrated in graded alcohol concentrations and embedded in paraffin. Paraffin sections of 5 μm thickness were cut, deparaffinized, and rehydrated. Sections were stained with hematoxylin/eosin (HE) (VWR/Thermo Fisher), Alcian Blue, and Picrosirius Red (PSR), in accordance to standard protocols, to analyze overall morphology and ECM components as GAG and collagen, respectively.

The distribution of collagen type II and collagen type I was visualized by immunohistochemistry (IHC). Heat induced antigen retrieval was performed using citrate buffer (pH 6,0, 2 × 5 min) at 90°C, followed by 10 min incubation with 3% H_2_O_2_ to block endogenous peroxidase activity. After 30 min blocking with a blocking solution (1% w/v Bovine Serum Albumin, 5% v/v normal rabbit serum, 0.2% v/v Tween 20), sections were incubated with monoclonal mouse anti-collagen II antibody for 2 h at RT (1:50, sc-518017, SantaCruz) or a monoclonal mouse anti-collagen I antibody (1:50, sc-293182, SantaCruz) at 4°C overnight. This was followed by a biotinylated rabbit anti-mouse antibody (1:200, E0413, Dako) as secondary antibody for 30 min. After washing, sections were treated with streptavidin-horseradish peroxidase for 30 min (1:200, Dako) after which 3,3-diaminobenzidine tetrahydrochloride (DAB, Sigma) served as a chromogen to visualize the coupled secondary antibody.

To assess proliferation, sections were stained with a mouse monoclonal antibody against the proliferation marker Ki67 (1:50, M7240, Dako). Staining procedure was similar as described above. The number of Ki67^+^ was manually counted and are reported in Supplementary Figure S1. All IHC stained sections were counterstained with Mayer’s hematoxylin and examined using an Olympus BX51 microscope. Sections were thoroughly screened and only images representative for the majority of the spheroids, hydrogels and scaffolds were selected.

Histomorphometric evaluation was performed to quantify the presence of the ECM components (GAG, collagen type I and type II). In brief, histological images (20× objective, Olympus BX51) were analyzed using the color deconvolution (“Alcian Blue and H” and “H DAB” vector) method in Image J. Automatic thresholding (RenyiEntropy method) was applied for the selection of GAG, collagen type I and collagen type II positive regions within spheroids. Positive stained area is shown as a ratio to the region of interest (ROI), i.e., total area of all spheroids on the slide. All measurements were performed in sixfold.

### Statistical Analysis

Data were analyzed using SPSS version 26.0 (SPSS GmbH Software) and are represented as the mean ± 95% confidence interval (CI). To test for normality of the variables, the Shapiro–Wilk test was used. The homogeneity of the variances was assessed with the Levene’s test. For data with a non-normal distribution, a Kruskal–Wallis test was performed. For the analysis of data with a normal distribution and homogeneous variances, a one-way ANOVA and Tukey’s *post hoc* test were performed. For data with a normal distribution with non-homogeneous variances, a Welch’s ANOVA test followed by a Games-Howell *post hoc* test were executed. For hydrogel properties, where two groups were compared and groups were normally distributed, an independent samples *t*-test was performed. *P* values < 0.05 were considered significant.

## Results

### Cartilage Microtissues

#### High-Throughput Formation of Cartilage Microtissues

**FIGURE 3:**
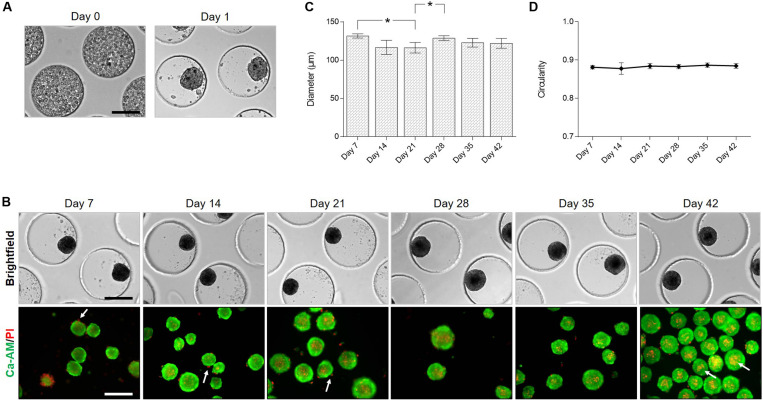
Formation of cartilage microtissues: morphology and viability. **(A)** hBM-MSC lowered spontaneously into the pores of the microwell. After 1 day of culture irregular shaped spheroids were formed. **(B)** Light microscopy and live/dead staining of spheroids over time in culture, white arrows indicate dead cells. **(C)** Evaluation of spheroid diameter and **(D)** circularity (*n* = 3, Welch’s ANOVA test followed by a *post hoc* Games-Howell, significant differences were marked as *p < 0.05). All data are presented as mean ± 95% CI. All scale bars represent 200 μm.

Spheroids were created using a non-adhesive agarose microwell system (Figure 1A). After seeding of the hBM-MSC suspension on the microwells, cells lowered into the bottom of the pores by gravitational force within 1–2 h and cells were distributed over the entire surface of the pore. Cells self-assembled spontaneously into slightly irregular shaped spheroids after 1 day of culture (Figure 3A). With increasing culture time, spheroids became more rounded and compact which is accompanied by a significant reduction in diameter from 131.68 ± 2.80 μm on day 7 to 116,28 ± 6.89 μm on day 21. Diameter slightly increased to a mean diameter of 122.09 ± 6.38 μm after 42 days of culture (Figure 3C). The circularity of the spheroids remained stable (±88%) (Figure 3D). Live/dead staining with calcein-AM/PI (Figure 3B) revealed high cell viability, cell dead is mostly observed at the periphery of the spheroids and in detached single cells. Few dead cells can be detected in the core of the spheroids, especially after 42 days of culture (indicated by white arrows, Figure 3B).

#### Proliferation and Maturation of Cartilage Microtissues

**FIGURE 4:**
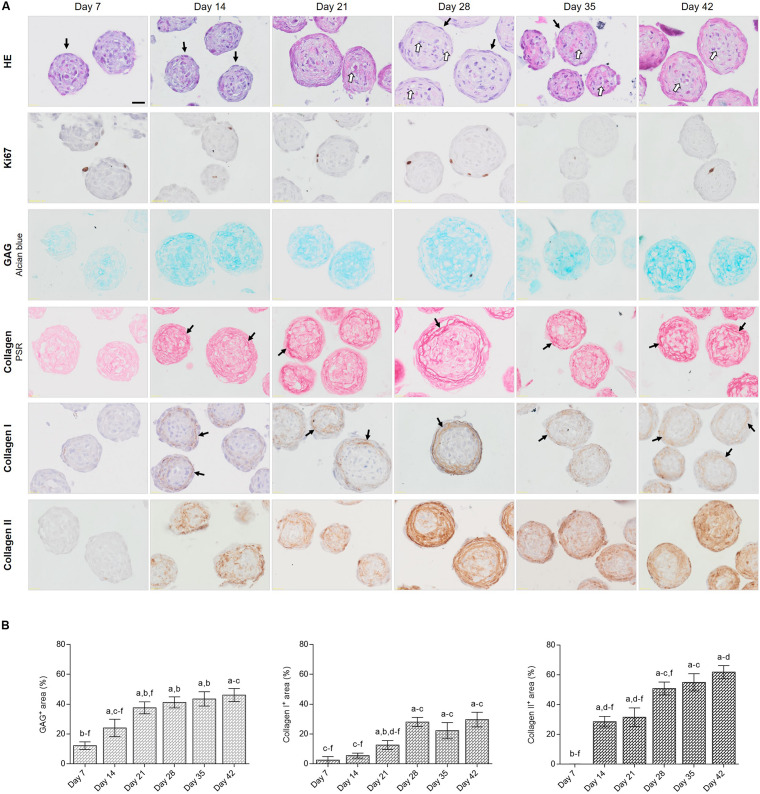
(Immuno)histological evaluation of cartilage microtissues: proliferation and extracellular matrix. **(A)** HE staining was performed to assess overall morphology, black arrows indicate flattened nuclei of cells at the periphery and white arrows indicate lacunae. IHC staining for Ki67 indicated proliferating cells, Alcian Blue staining showed the presence of glycosaminoglycans (GAG). Collagen was visualized by Picrosirius Red (PSR) staining, black arrows indicate mature fibrillar collagens. IHC stainings for collagen type I and type II were performed, black arrows indicate collagen type I fibers. Scale bar 20 μm. **(B)** Quantification of GAG, collagen I and collagen II positive stained areas. All data are presented as mean ± 95% CI. Significant differences (*p* < 0.05) were marked ^a^ compared to day 7, ^b^ to day 14, ^c^ to day 21, ^d^ to day 28, ^e^ to day 35, and ^f^ to day 42 (*n* = 6, One-way ANOVA followed by a Tukey’s *post hoc* test).

Histological analysis was performed to screen overall morphology (HE), proliferation (Ki67), and cartilage ECM (Figure 4). HE staining shows the presence of nuclei throughout the spheroid. Nuclei appeared more flattened at the periphery of the spheroid (indicated by black arrows, Figure 4A). Spheroids displayed a cartilage-like morphology with lacunae with increasing culture time (indicated by white arrows, Figure 4A).

Proliferation within the spheroids was evaluated by performing an IHC staining against the proliferation marker Ki67. Staining showed that Ki67 positive cells were mostly located in the outer rim of the spheroids (Figure 4A). The highest number of Ki67^+^ cells was reported after 7 days of culture (2.14 ± 0.85 cells/spheroid) (Supplementary Figure S1). On day 28, spheroids presented a significant higher number of Ki67 positive cells (1.31 ± 0.55 cells/spheroid) as compared to day 21 (0.31 ± 0.30 cells/spheroid), which correlates with the increase in diameter (Figure 3C). The number of Ki67 positive cells reduced after 35 and 42 days in culture (0.17 ± 0.26 and 0.08 ± 0.20 cells/spheroid, respectively (Figure 4A and Supplementary Figure S1).

To induce chondrogenic differentiation of hBM-MSC spheroids to mature cartilage microtissues, serum-free chondrogenic culture medium and low oxygen tension (5%) were applied. As the goal of our study is to develop articular cartilage-like microtissues, composition and distribution of cartilage specific ECM components was extensively screened. GAG were visualized by Alcian Blue staining. From day 7, slightly positive staining for GAG was detected (12.16 ± 2.57%), especially in the spheroid core. With increasing culture time, positive stained area and staining intensity both increased (46.13 ± 4.36% on day 42), resulting in the presence of GAG covering the entire spheroid area (Figures 4A,B). Picrosirius Red staining showed slightly positive collagen fibers in the 7-day-old spheroids and from 14 days in culture, mature fibrillar collagen was demonstrated (black arrows, Figure 4A). Starting from day 21, a clear presence of fibers especially located at the periphery of the spheroid was reported (black arrows, Figure 4A). This corresponds to the IHC localization of collagen type I, where fibers showed to be present exclusively in the outer rim of the spheroids, covering 2.49 ± 2.36% of the spheroid area after 7 days of culture and increased to 29.64 ± 4.90% after 42 days of culture (Figures 4A,B). This is in contrast to the distribution of collagen type II, which is more dispersed throughout the entire spheroid. Collagen type II manifested starting from 14 days of culture, mainly situated at the periphery covering 28.56 ± 3.52% of spheroid area, and the positively stained area increased with increasing culture time, stretching toward the center covering 61.83 ± 4.40% of the spheroid area on day 42 (Figures 4A,B). Some spheroids are devoid of collagen type II in the center but Alcian Blue staining shows the presence of GAG in the core of the spheroid (Figure 4A).

### Cartilage Microtissue Fusion Into a Macrotissue

**FIGURE 5:**
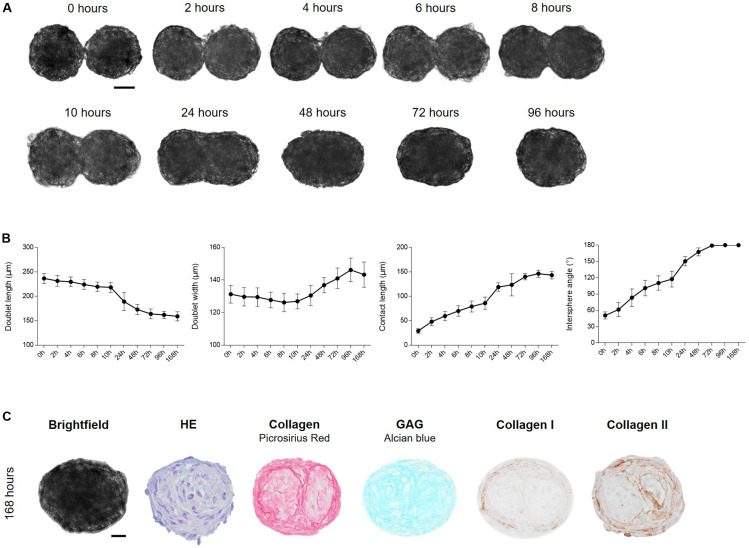
Fusion of cartilage microtissue doublets. **(A)** Cartilage microtissues at maturation stage of 7 days were harvested and doublets were formed. Spheroid fusion was tracked for 168 h by light microscopy. Scale bar 50 μm. **(B)** Morphological parameters such as doublet length (μm), doublet width (μm), contact length (μm), and intersphere angle (°) were measured using Image J software. (*n* = 12, all data are presented as mean ± 95% CI). **(C)** Morphological and (immuno)histochemical evaluation of fused doublets after 168 h of culture. HE staining was performed to assess overall morphology, Alcian Blue staining showed the presence of glycosaminoglycans (GAG). Collagen was visualized by Picrosirius Red (PSR) staining and IHC stainings for collagen type I and type II were performed. Scale bar 20 μm.

To investigate the potential and time frame of immature 7-day-old cartilage microtissues to fuse, spheroids were placed in close proximity to each other by performing a doublet formation assay (Figure 2; Susienka et al., 2016). For the creation of spheroid doublets, the donor microwell was cropped and inversely stacked on the recipient microwell. After centrifugation spheroids of the donor microwell lowered into the wells of the recipient microwell, resulting in pairwise placement of spheroids, creating doublets (Figure 5A). Microwells comprising triplets and quadruplets were excluded for evaluation. During fusion, doublets rotated both clock- and counterclockwise in the pores of the microwell. To analyze the time span of fusion of a mature cartilage microtissue, morphological changes during the first hours of the fusion process were imaged. Doublet length, doublet width, intersphere angle and contact length between spheroids were measured. Initial contact between spheroids occurs within the first hour (Figure 5A). As fusion progressed, doublet lengths shortened in function of time starting from 236.53 ± 10.24 μm to 158.91 ± 9.41 μm after 168 h. Intersphere angle and contact length increased in function of time at a similar rate. After 72 h of fusion, the intersphere angle reached a plateau (±179°) and did not change anymore in function of prolonged culture time. Contact length approached 140 μm which matches the width of the spheroids, indicating complete spheroid fusion. However, contact length and doublet width showed some minor deviations indicating continuous dynamic processes in the fused spheroids. The fused spheroid after 96 h indeed shows a more rounded morphology compared to the oval spheroid after 72 h (Figures 5A,B). Histological evaluation of the fused doublet after 168 h is shown in Figure 5C. Although HE staining shows a perfectly fused doublet, ECM stainings (PSR, Alcian Blue, collagen I, and II) clearly show the margins of the individual cartilage microtissues. Especially collagen type I (demonstrated by PSR and IHC) is situated at the periphery of the individual microtissues and the fused doublet (Figure 5C). The newly synthesized matrix surrounding the original individual spheroids is mainly composed of collagen II (Figure 5C).

**FIGURE 6:**
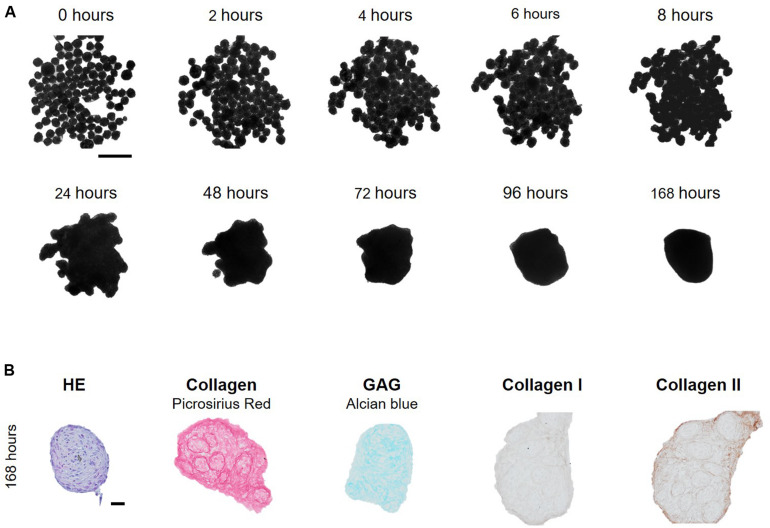
Formation of a macrotissue by fusion of multiple cartilage microtissues. **(A)** Cartilage microtissues at maturation stage of 7 days were harvested and ± 200 spheroids were seeded per agarose coated 96-well. Spheroid fusion was tracked for 168 h. Scale bar 500 μm. **(B)** Morphological and (immuno)histochemical evaluation of the fused microtissues after 168 h of culture. HE staining was performed to assess overall morphology, Alcian Blue staining showed the presence of glycosaminoglycans (GAG). Collagen was visualized by Picrosirius Red (PSR) staining and IHC stainings for collagen type I and type II were performed. Scale bar 20 μm.

To confirm if a large number of spheroids was able to fuse into a macrotissue, ±200 7-day-old spheroids were seeded in agarose coated 96-wells (Figure 6). Spheroids made contact within 2 h after seeding. After 24 h, margins between spheroids faded and no individual spheroids could be distinguished. Further compaction occurred and after 96 h, a circular construct of ±527 μm diameter was formed (Figure 6A). After 168 h of fusion, histology showed the presence of GAG throughout the entire fused tissue, collagen I and especially collagen II were present at the periphery of the fused tissue and at the periphery of the original individual spheroids, which still can be perceived with histology (Figure 6B).

### Encapsulation of Cartilage Microtissues in gelMA

Because spheroids started to developed a cartilage ECM after 14 days of culture, as demonstrated by GAG (24.06 ± 5.84%) and collagen type II content (28.56 ± 3.52%) (Figures 4A,B), spheroids at this maturation stage were selected to encapsulate in gelMA. To assess the impact of the biopolymer and PI on spheroid morphology, viability, phenotype, fusion and outgrowth, spheroids were encapsulated in hydrogel disks of 10 w/v% gelMA. Polymerization by UV-light was performed with 2 mol% Irgacure 2959 or Li-TPO-L and their effect on hydrogel properties was assessed as well.

#### Morphology, Viability and Fusion of Encapsulated Cartilage Microtissues

**FIGURE 7:**
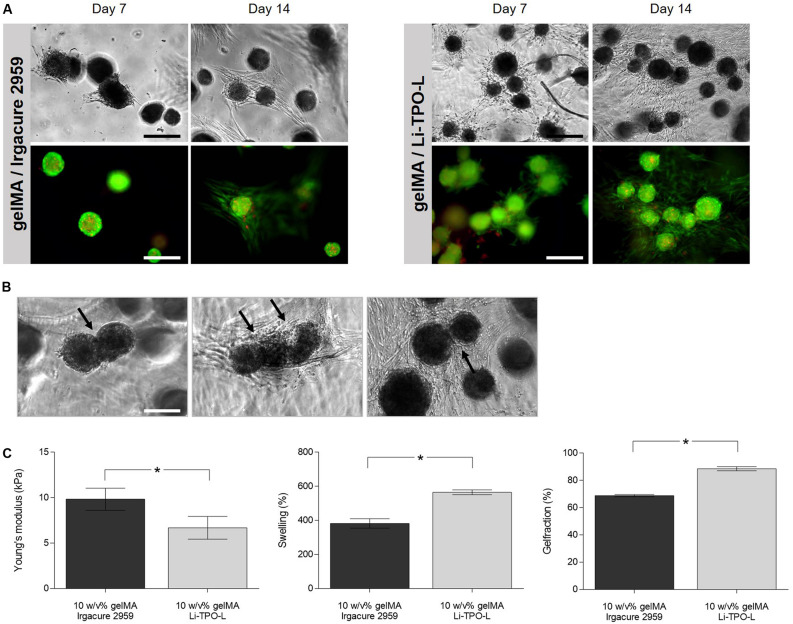
Influence of gelMA properties on spheroid morphology, viability, and fusion. **(A)** Cartilage microtissues at maturation stage of 14 days were harvested and were encapsulated in gelMA using either Irgacure 2959 or Li-TPO-L as a photo-initiator. Morphology and viability of encapsulated spheroids 7 and 14 days post encapsulation. Scale bars 200 μm. **(B)** Cellular outgrowth and fusion of 14-day-matured cartilage microtissues within gelMA after 14 days of culture in the hydrogel, fused spheroids are indicated by the black arrows. **(C)** Young’s modulus, gel fraction and swelling ratio were assessed on cell-free gelMA hydrogel samples (*n* = 5, independent samples *t*-test). All data are presented as mean ± 95% CI and significant differences were marked as *p < 0.05.

Spheroids encapsulated in gelMA displayed a round morphology (Figure 7). In the gelMA/Irgacure2959 hydrogels, cellular outgrowth was observed after 7 days. In contrast, when Li-TPO-L was used as a PI, more cellular outgrowth was observed after 7 and 14 days post encapsulation. After 14 days, cellular sprouts were spread within the entire hydrogel disk. Live/dead staining showed that spheroids and their cellular outgrowths remained viable in all hydrogels, regardless the PI used for photopolymerization (Figure 7A). Encapsulated spheroids located in near vicinity of each other still have the capacity to fuse, as indicated by the black and white arrows in Figure 7B. Nevertheless, spheroid fusion post encapsulation is strongly dependent on the position of the spheroids within the hydrogel and progresses slower. Only incomplete fusion is reached during the 14-day time frame of the encapsulation experiment, reaching an intersphere angle of ±127°. This is in contrast with the non-encapsulated spheroid doublets where complete fusion (intersphere angle of ±180°) is obtained after 72 h.

#### Physico-Chemical Properties of GelMA

The mechanical and physico-chemical properties including Young’s modulus, gel fraction and swelling behavior were evaluated for cell-free gelMA hydrogel disks, crosslinked in the presence of Irgacure 2959 or Li-TPO-L (Figure 7C). Irgacure 2959 as PI resulted in samples with significantly higher Young’s moduli (9.84 ± 1.22 kPa) compared to Li-TPO-L samples (6.69 ± 1.25 kPa).

The results of the swelling test indicate that all conditions were able to absorb large quantities of water, which mimics the content of native cartilage tissues as cartilage is mainly composed of water (80%). Li-TPO-L crosslinked hydrogels showed significantly higher swelling (565.1 ± 13.87%) compared to Irgacure 2959 crosslinked hydrogels (382.59 ± 27.78%). Irgacure 2959 crosslinked hydrogels had a lower gel fraction (68.84% ± 0.81) compared to Li-TPO-L crosslinked hydrogels (88.61% ± 1.48).

#### Influence of GelMA on Proliferation and Cartilage Phenotype

**FIGURE 8:**
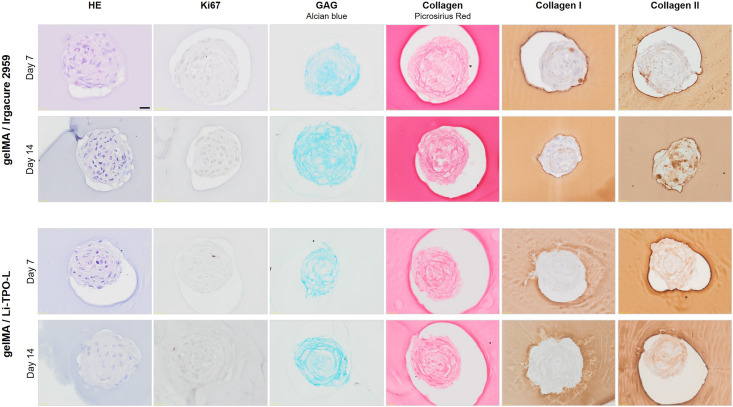
Evaluation of cartilaginous phenotype 7 and 14 days post encapsulation in gelMA. Comparison of Irgacure 2959 or Li-TPO-L as a photo-initiator. HE staining was performed to assess overall morphology, IHC staining for Ki67 indicated proliferating cells, Alcian Blue staining showed the presence of glycosaminoglycans (GAG). Collagen was visualized by Picrosirius Red (PSR) staining and IHC stainings for collagen type I and type II were performed. Scale bar 20 μm.

Hematoxylin/eosin staining showed that the hydrogel encapsulation process did not change the morphology of the spheroids. In contrast, immunohistochemical staining for Ki67 demonstrates that encapsulated cells rarely proliferated, only few positive cells were found (0.29 ± 0.49 and 0.43 ± 0.53 cells/spheroid after 7 and 14 days of encapsulation, respectively) (Figure 8).

**FIGURE 11:**
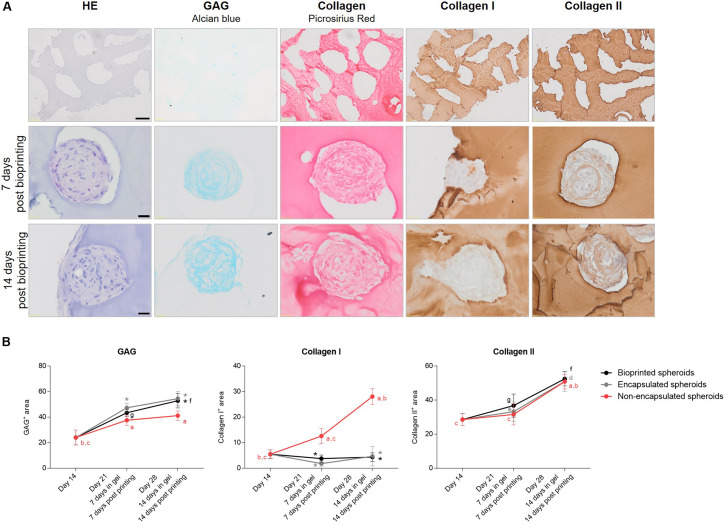
Evaluation of cartilaginous phenotype of microtissues post printing. **(A)** HE staining was performed to assess overall morphology, Alcian Blue staining showed the presence of glycosaminoglycans (GAG). Collagen was visualized by Picrosirius Red (PSR) staining and IHC stainings for collagen type I and type II were performed. Scale bar 20 μm. **(B)** Graphs represent the quantification of GAG, collagen I and collagen II positive stained areas within bioprinted spheroids as compared to encapsulated spheroids in gelMA/Li-TPO-L and non-encapsulated spheroids from corresponding culture timepoints. All data are presented as mean ± 95% CI. For non-encapsulated spheroids, significant differences (*p* < 0.05) between culture timepoints were marked ^a^ compared to day 14, ^b^ to day 21, and ^c^ to day 28 spheroids. For encapsulated spheroids significant differences (*p* < 0.05) between culture timepoints were marked ^d^ compared to 7 days post encapsulation and ^e^ to 14 days post encapsulation. For bioprinted spheroids significant differences (*p* < 0.05) between culture timepoints were marked ^f^ compared to 7 days post printing and ^g^ to 14 days post printing. For both encapsulated (in gray) and bioprinted spheroids (in black) significant differences compared to the non-encapsulated spheroid controls were marked * (*n* = 6, One-way ANOVA followed by a Tukey’s *post hoc* test).

Histological analysis was performed to assess the effect of gelMA encapsulation on the cartilaginous phenotype of the spheroid. Alcian Blue staining showed the presence of GAG which increased with extending culture time, though not significantly, from 47.33 ± 3.46% 7 days post encapsulation to 54.52 ± 5.47 14 days post encapsulation (Figures 8, 11B). Moreover, combination with gelMA seems to have a positive effect on GAG production, indicated by a significantly higher positively stained area by Alcian Blue staining in the encapsulated spheroids (Figure 8) as compared to the non-encapsulated spheroids (37.59 ± 4.00% and 41.19 ± 3.69% on day 21 and 28, respectively) (Figures 4, 11B). For the evaluation of collagen content, PSR, collagen type I, and type II stainings were performed. Once encapsulated in gelMA, only slightly positive staining for collagen type I was observed. This reduction in collagen type I content in encapsulated spheroids (1.81 ± 1.01% 7 days post encapsulation and 4.68 ± 3.78% 14 days post encapsulation) was significant as compared to non-encapsulated controls (12.57 ± 2.97% and 28.04 ± 3.05% on day 21 and 28, respectively) (Figure 11B). Spheroids in gelMA/Li-TPO-L hydrogels showed a reduced presence of collagen type I as compared to gelMA/Irgacure 2959 hydrogels. Moreover, spheroids encapsulated in gelMA/Li-TPO-L hydrogels had a higher and more dispersed collagen type II content (Figure 8). Collagen type II content was not affected by encapsulation (Figure 11B). Therefore, this condition was selected for further experiments.

### Extrusion Bio-Ink Based 3D Bioprinting of Cartilage Microtissues

**FIGURE 9:**
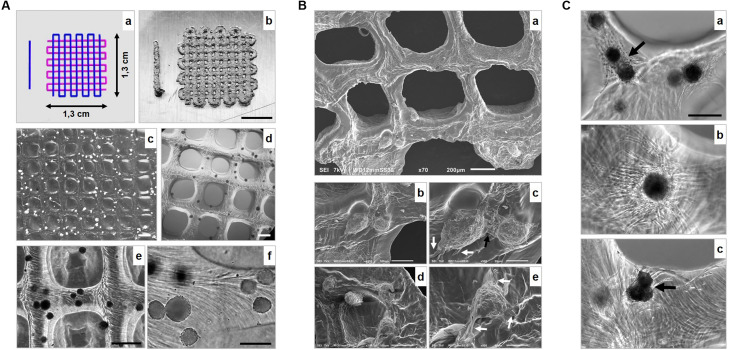
3D bioprinting of 14-day-mature cartilage microtissues in gelMA/Li-TPO-L **(A)** Representation of **(a)** the CAD model and **(b–f)** macroscopic to microscopic representation of the printed scaffolds. Scale bars 500 μm **(b)**, 800 μm **(c)**, 500 μm **(d,e)**, 200 μm **(f)**. **(B)** Scanning Electron Microscopy (SEM) evaluation of bioprinted scaffolds. Black arrow indicates cellular sprouts of adjacent spheroids making contact, initiating fusion **(b)** and white arrows **(b,c)** indicate cellular outgrowth. Scale bar 200 μm **(a)**, 100 μm **(b,d)**, 50 μm **(c,e)**. **(C)** Fusion and cellular outgrowth of spheroids 4 days **(a)** and 14 days **(b,c)** post printing within the gelMA. Black arrows indicate tissue fusion. Scale bar 200 μm.

The processing potential of the bio-ink, consisting of gelMA/Li-TPO-L containing cartilage microtissues at maturation stage of 14 days was evaluated using extrusion-based 3D bioprinting (Figure 9). Processing parameters such as ambient temperature, cartridge temperature, needle type, and diameter, were optimized (Table 1).

**FIGURE 10:**
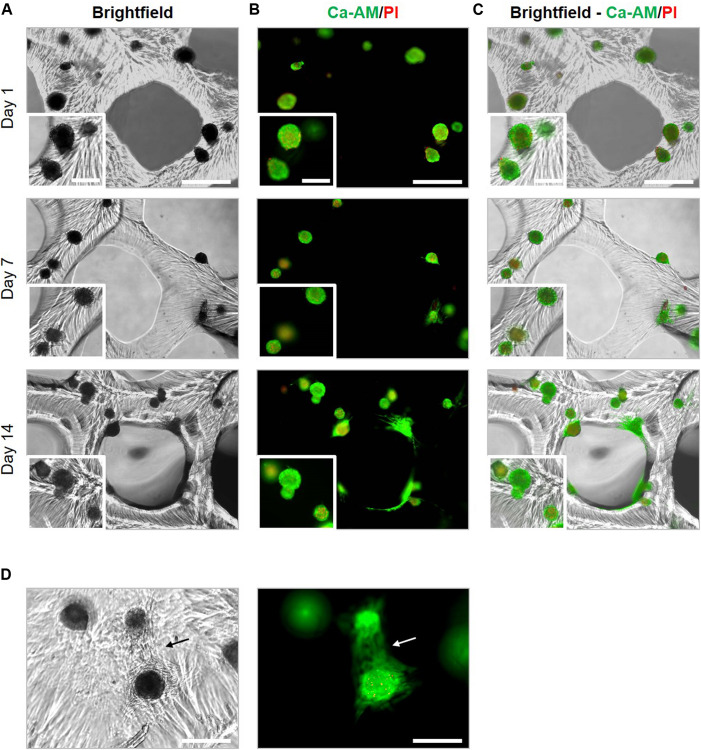
Viability of cartilage microtissues post 3D bioprinting. The viability of spheroids bioprinted at maturation stage of 14 days was assessed by live/dead staining with ca-AM/PI after 1, 7, and 14 days post printing. **(A)** Brightfield images, **(B)** fluorescent images of ca-AM/PI staining and **(C)** merged brightfield and ca-AM/PI images. Scale bars 500 μm (overview) and 200 μm (insert). **(D)** Cell outgrowth within printed constructs 14 days post printing. Cells spreading out from a surfacing spheroid (lower spheroid) toward a deeper situated spheroid (upper spheroid). Scale bar 500 μm.

Scaffolds (1.3 × 1.3 cm) composed of four layers in a 0/90° lay-down pattern were printed according to a computer-aided design (CAD), using an applied pressure of approximately 0.035 MPa [Figure 9A(a)]. Scaffolds displayed highly controlled macropore morphology, with pore sizes around 800 μm and struts with a mean thickness of 480.01 ± 20.90 μm (measured after 30 min immersion in culture medium post printing). Spheroids were dispersed throughout the entire scaffold [Figure 9A(b–f)]. SEM analysis was performed after 4 days of culture to investigate scaffold and spheroid morphology (Figure 9B). The dehydration process for SEM analysis caused shrinkage of the scaffold samples. SEM of the gelMA showed an organized networked structure. Spheroids show cellular outgrowth [white arrows, Figure 9B(c,e)] and when placed adjacent to each other, cellular sprouts made contact, initiating spheroid fusion [black arrow, Figure 9B(c)]. Brightfield images after 4 and 14 days of culture (Figure 9C) confirmed that after the printing process, spheroids were still able to grow out, and that is, dependent on their initial position, either on the outer surface of the printed construct or deeper into the hydrogel (Figure 10D). When deposited in close proximity to each other, spheroids were able to fuse in the printed construct [indicated by black arrows, Figure 9C(a,c)]. Culturing of the bioprinted scaffolds for 14 days, showed that scaffolds retained their 3D structure and that spheroids remained at their initial 3D spatial position (Figure 10A). The printing process did not affect spheroid viability as observed by live/dead staining (Figures 10B–D). 14 days post printing, spheroids and sprouts of nearly surfacing, partially protruding and fully encapsulated spheroids were still viable (Figures 10B–D). However, complete fusion of cartilage microtissues was not obtained yet.

**FIGURE 12:**
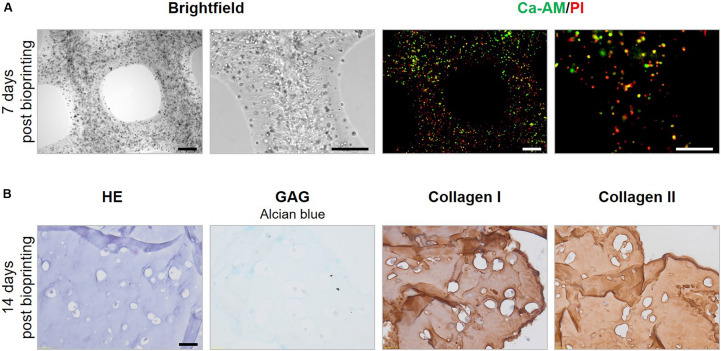
Evaluation of bioprinted single cells. **(A)** Morphology and viability of bioprinted single cells 7 days post printing. Scale bars 200 μm. **(B)** HE staining was performed to assess overall morphology, Alcian Blue staining showed the presence of glycosaminoglycans (GAG) and IHC stainings for collagen type I and type II were performed. Scale bar 20 μm.

Histological evaluation and macroscopic pictures confirmed the 3D printed pattern composed of struts and pores (Figure 11, first row). Microscopical histological evaluation showed that post printing spheroids displayed good morphology with intact nuclei throughout the entire spheroids. More importantly, spheroid ECM was not altered or did not deteriorate after enduring the printing process. 7 days post printing, spheroids displayed high GAG and collagen type II content, and low to no collagen type I presence, which is in line with the results of the encapsulation experiments, no significant differences were observed for the ECM components in comparison with the encapsulated spheroids (Figures 11A,B). Moreover, GAG and collagen type II content increased over time in culture from 43.40 ± 2.75% to 52.82 ± 5.63% and 36.79 ± 6.75% to 52.45 ± 4.15%, respectively, 7 and 14 days post printing (Figure 11B). Bioprinting of spheroids clearly outperformed the bioprinting with single cells (Figure 12). Constructs with single cells showed poor viability 7 days post printing and no chondrogenic differentiation was observed after 14 days. Spheroids seem to withstand the printing process better, probably because of their robust ECM.

## Discussion

Cell-based regenerative therapies for cartilage repair and regeneration are trending, with (M)ACI as a well-known example. However, this procedure often results in the creation of reparative tissue, with inferior quality and lacking durability when compared to the native tissue. This can be attributed to chondrocytes having the tendency to dedifferentiate during expansion in 2D culture (Dewan et al., 2014; Deng et al., 2016; Onofrillo et al., 2018; De Moor et al., 2019). Moreover, the application of individual cells fails to mimic the complex cell-cell and cell-matrix interactions present within a 3D tissue, which hinders the accurate imitation of the early events in tissue development (Gionet-Gonzales and Leach, 2018).

In the search for an alternative to freely inject or transplant cells into the defect site, 3D bioprinting holds a great promise to fabricate a tissue construct mimicking the native tissue by the directed assembly of microscale building blocks into a larger tissue construct. We explored a hybrid strategy combining cellular microtissues, biomaterials and 3D printing technology. For the first time, small spheroids of BM-MSC were generated using a cost-effective system and the potential of a low-cost modified version of the natural polymer gelatin (gelMA) was tested to serve as an instructive ink for cartilage microtissues to create a macroscale tissue. This work aimed to elucidate if BM-MSC spheroids are able to differentiate into cartilage microtissues and how they respond to encapsulation in a gelMA hydrogel environment. The promise of this approach is that in future studies cartilage microtissues of different maturation stages mimicking endochondral ossification could be printed in a stratified pattern resembling osteochondral interfaces. This stratified approach cannot be obtained by current clinical therapy with injecting of cells or spheroids (chondrospheres).

Prior to encapsulation of spheroids with gelMA, different culture times were evaluated to select the maturation stage of the cartilage microtissue (optimal spheroid diameter and ECM content). Self-assembled 3D cartilage microtissues from human BM-MSC were successfully generated using a non-adhesive agarose microwell system. This high-throughput technique allowed the controlled formation of spheroids with a mean diameter of 116.73 μm after 14 days of culture, which is an optimal geometry for deposition by the print needle. 3D microtissues can be created in several other ways, using pellet culture, spinner flask culture, hanging drop method, etc., the main disadvantages of these techniques are the heterogeneity of the spheroids (intervariability in dimensions is reflected in suboptimal chondrogenic differentiation), the quantity of the produced spheroids and the intensive manual labor for the exchange of culture medium and spheroid harvesting (Achilli et al., 2012).

In this study, the spheroids were homogeneous in shape and size, crucial for efficient deposition by 3D bioprinting. In addition to homogeneity and stability in diameter and circularity along the culture period, spheroids showed a high viability during chondrogenesis, which started from 14 days in culture, indicated by the manifestation of cartilage ECM components such as GAG and collagen II. Histological analysis also showed that GAG and collagen II increased over the 42 days of spheroid culture time. Chondrogenesis was induced by a low oxygen culture environment, mimicking the physiological concentration and the application of a serum-free chondrogenic culture medium containing TGF-β1. It has been described that TGF-βs contribute in the regulation of chondrogenic differentiation from the early to terminal stages, including condensation, proliferation, terminal differentiation and maintenance of articular chondrocytes. More specifically, the TGF-β1 signaling pathway induces mesenchymal condensation via up-regulation of N-cadherin and fibronectin and TGF-β1 treatment initiates chondrogenesis of mesenchymal progenitor cells (Wang et al., 2014). In this regard, it is important that spheroid cultures have a low variability in spheroid dimensions allowing to homogeneously differentiate into the cartilage phenotype and leading to thousands of cartilage microtissues at the same maturation stage.

Hydrogels have been widely used as 3D matrices for cell application. They are used as a medium for the retention of cells after injection in the cartilage defect site or as a carrier for cells or spheroids during bio-ink based extrusion printing to ensure shape fidelity of the construct (Moldovan et al., 2018; Li et al., 2019). Common biomaterials used in cartilage engineering are collagen, alginate, hyaluronic acid, etc., because of their resemblance with the native tissue ECM (Li et al., 2019). In this work gelMA was chosen because it has shown to be a permissive environment for neo-cartilage formation when cell suspensions of chondrocytes or MSC are encapsulated (Levato et al., 2017; Rothrauff et al., 2017). After encapsulation, cartilage microtissues proved to retain their geometry and although the crosslinking process involving UV-light could be detrimental for cell viability, it was preserved in the hydrogel. However, a shift in proliferative capacity was observed after embedding in gelMA, limited Ki67 positive cells were found in comparison to non-encapsulated spheroids. Although this lack of Ki67 staining, light microscopic evaluation showed the cellular outgrowth. Similar results were reported when spheroids of human adipose-derived MSC were encapsulated in 5, 7.5, and 10 w/v% gelMA hydrogels: sprouting was observed and was more pronounced in the softest gels (Žigon-Branc et al., 2019). In this study, outgrowth of cells was especially seen in the softer gelMA hydrogels crosslinked with Li-TPO-L as a PI. Interestingly, these hydrogels with a significantly lower Young’s modulus did display a higher gel fraction than hydrogels crosslinked with Irgacure 2959. The latter is expected because a LED with higher intensity was used to crosslink the Li-TPO-L gels and Li-TPO-L has an absorption maximum at approximately 375 nm. Therefore, at the wavelength of 365 nm, it provides better reactivity compared to Irgacure 2959, whose absorption already tails out in this spectral range and Li-TPO-L also exhibits a higher efficiency and yield of radical formation (Markovic et al., 2015). It is hypothesized that crosslinking with Li-TPO-L probably results in a more homogenously distributed network throughout the entire hydrogel, as compared to Irgacure 2959 which possibly results in a more heterogeneously distributed crosslinked network with initial crosslinking at the surface and less transmission of the UV-light deeper in the gel, explaining the higher mechanical strength but lower gel fraction. Moreover, different crosslinking times and light sources with different intensities were used to crosslink the Irgacure 2959 and Li-TPO-L gels, respectively, explaining the differences in mechanical properties and gel fraction.

Regardless of the hydrogel stiffness, the encapsulated chondrogenic induced spheroids presented a GAG- and collagen type II-rich ECM, increasing with time in culture in the hydrogel. It was already described by Salamon et al. (2014) that gelMA had a pro-chondrogenic impact on MSC. When adipose-derived MSC were seeded on top of gelMA films, an upregulation of Sox9, Sox5, and Col2a1 gene expression was described (Salamon et al., 2014). Moreover, seeding adipose-derived MSC on gelMA resulted in a stronger accumulation of GAG compared to controls (Salamon et al., 2014). We also observed that gelMA encapsulation had a cumulative effect on GAG production within spheroids and Roosens et al. (2019) reported the same trend when spheroids consisting of valvular interstitial cells were encapsulated. The constructs with lower stiffness resulted in a downregulation of collagen I and an increase in collagen II production. Žigon-Branc et al. (2019) also demonstrated that the extent of chondrogenic differentiation of MSC spheroids was more pronounced in softer hydrogels.

The bioprinting processability of spheroid-laden gelMA/Li-TPO-L bio-ink was improved after optimization of the printhead heater and environmental temperature. Hence, gelMA scaffolds with encapsulated cartilage microtissues demonstrated good shape-fidelity. Moreover, the printed scaffolds were stable for the entire culture period and spheroid viability and phenotype remained after enduring the printing process. For the creation of large scale constructs with relevant mechanical strength, the hydrogel can be reinforced with a synthetic polymer such as polycaprolactone (PCL), a popular polymer due to its superior rheological and viscoelastic properties (Woodruff and Hutmacher, 2010). Daly et al. (2016) co-deposited hydrogel bio-inks with PCL filaments and PCL-reinforced hydrogel scaffolds had compressive moduli (2 MPa) similar to that of native articular cartilage. Co-depositioning with PCL fibers can lead to stress shielding if the young’s modulus of the implant is higher than the modulus of the surrounding tissue which will lead to the PCL fibers carrying most of the externally applied forces. It is important to keep this strain mismatch between implant and surrounding tissue limited, preventing poor implant performance and surrounding tissue damage (Wintermantel et al., 2001; Farag, 2017).

Recently, a material-free assembly method was used to create a cartilage construct starting from spheroids (Grogan et al., 2019). The Kenzan bioprinting method does not use any material support structure, but relies on natural cell-to-cell contact behavior and spheroid fusion after fixation upon microneedles that provide temporary support (Moldovan et al., 2017). Spheroids used for this method are characterized by a large diameter (>500 μm) (Grogan et al., 2019). However, creating viable tissues with large diameter is challenging due to the diffusion limit (100–200 μm) (Radisic et al., 2006; Liu et al., 2015). Especially centrally located cells of an engineered construct, lack sufficient supply of nutrients and oxygen, and are unable to dispose metabolic products (e.g., CO_2_), causing poor viability (Alvarez-Pérez et al., 2005; Gaskell et al., 2016). For this reason, we opted to start from small diameter spheroids. Their ability to fuse is essential in this synergistic hybrid tissue engineering approach, as tissue fusion is the fundamental biological process to build larger structures. When two spheroids were placed together, creating a doublet, cartilage microtissue fusion was completed after 72 h of culture as indicated by reaching a plateau of the intersphere angle. During the first 24 h of fusion, doublet length and width decreased, suggesting the compaction process. Doublet length further decreased but doublet width started to increase, approaching the measurements of the doublet length indicating that doublets started to compact and dynamically reorient to a circular shape. The fused doublet displayed cartilage specific molecules, GAG and collagen II, GAG were dispersed throughout the fused doublet, while collagen II was mainly present in the newly synthesized matrix surrounding the original individual spheroids. Doublet fusion of primary chondrocyte spheroids was already described by Parfenov et al. (2018) and Susienka et al. (2016) Both studies showed that sheep and human primary chondrocyte spheroids/doublets, respectively, were able to fuse (Susienka et al., 2016; Parfenov et al., 2018). Our study investigated, for the first time, fusion and morphological changes during chondrogenic induced BM-MSC doublet formation.

Doublet fusion could be transferred to fusion of multiple spheroids in an agarose coated well. A compact macrotissue was created after 96 h which showed increased circularity over time in culture. Notably, there was no presence of collagen type II in the center of the fused construct, this can be explained by the use of immature day 7 spheroids, where there is no manifestation of ECM, and the limited diffusion of nutrients into the core of the macrotissue. Spheroid fusion was still observed when spheroids were encapsulated in gelMA hydrogels in close proximity to each other, demonstrating the tissue-fusion-permissive potential of the hydrogel. This was confirmed after completing the printing process where spheroids within the scaffold structure were still able to fuse. However, we want to highlight the time discrepancy between spheroid fusion in the absence versus in the presence of a hydrogel. Complete fusion of spheroids is obtained after 72 h in absence of a hydrogel, as indicated by an intersphere angle of ±179°. When spheroids are encapsulated or bioprinted in gelMA, fusion is highly dependent on a close deposition of neighboring spheroids. Encapsulated spheroids in gelMA did not completely fuse during the 14-day time frame of the experiment, shown by an intersphere angle of 127°. This was in part due to the difference in age of the used spheroids, the spheroids used in the non-encapsulated fusion experiments were 7-day-old spheroids, while 14-day-matured spheroids were encapsulated or bioprinted. It has been described that fusion of older spheroids results in incomplete fusion showing the individual margins of the spheroids (De Moor et al., 2018). Nevertheless, while hydrogel degradation will occur *in vivo*, spheroids will have enhanced probability to fuse. This underlines the importance of research dedicated to find hydrogels or biomaterials with altered characteristics having the capability to enhance the functionality of bioprinted tissues. This field of smart biomaterials research will definitely have a positive impact on the biofabricated tissues.

Traditional ACI procedures using a cell suspension of chondrocytes, or MACI procedures using cells in combination with a scaffold, are indicated for large femoral defects of 2–10 cm^2^ and 2 million autologous cells/cm^2^ are applied (Foldager et al., 2012; Becher et al., 2017; Krill et al., 2018). Only 12–26 weeks after implantation of the single cell suspension, cartilage matrix develops (Becher et al., 2017; Krill et al., 2018). The major advantage of using spheroids instead of single cells is that, because of the high cell-cell contacts and cell density in 3D preculture, they already have developed a tissue specific ECM, before they are used for implantation (Bartz et al., 2016; De Moor et al., 2019). We believe the use of spheroids will lead to a less fibrous tissue than traditional ACI because they already possess a GAG- and collagen II-rich ECM. Moreover, spheroids combined with gelMA also displayed a significantly less fibrous and a more hyaline cartilage-like phenotype. Interestingly, bioprinting of spheroids seems to be more successful than bioprinting of single cells since constructs containing single cells showed poor viability, and more importantly no chondrogenic differentiation (Figure 12). When using spheroids as building blocks, the concentration of spheroids in the bio-ink needs to be optimized. One bioprinted construct of four layers was printed, with dimensions of 1.3 cm × 1.3 cm and thus covering an area of 1.69 cm^2^, using ±167 μl of bio-ink. The concentration of spheroids in the bio-ink was 12680 spheroids/ml, thus 1 construct contained ±2113 spheroids/1.69 cm^2^ which equals 1250 spheroids/cm^2^. We suggest to increase the concentration in accordance with the clinically applied cell concentration in traditional ACI (2 million cells/cm^2^), this corresponds with 4 seeded microwells and thus 6340 spheroids/cm^2^. To achieve a similar concentration as in traditional ACI, the spheroid concentration in the bio-ink needs to be a fivefold higher (±63400 spheroids/ml, corresponding with 40 microwells). Enhanced fusion of spheroids will be the result of the increased spheroid concentration. *In vivo*, the gelMA scaffold will be prone to degradation, leading to softer hydrogels with impact on nutrient diffusion, spheroid fusion and cellular outgrowth.

Our study shows that 3D cartilage microtissues can be bioprinted while maintaining cell viability, 3D architecture, chondrogenic phenotype and their fusion capacity. The versatile fusion capacity of microtissues is interesting for future *in vivo* implantation as this shows the broad spectrum of application possibilities. Spheroids can be used as such, or in combination with gelMA as an injectable. Highly interesting is that a patient-specific implantable construct could be manufactured by 3D bioprinting of chondrogenic induced spheroids at various endochondral developmental stages giving the promise of treating stratified osteochondral defects.

## Data Availability Statement

The datasets generated for this study are available on request to the corresponding author.

## Author Contributions

LD and HD: participation in manuscript writing, study design, study performance, and data analysis. SF: performance of the experiments. MA and ND: scanning electron microscopy. LT, CV, SV, and PD: synthesis of gelMA and data interpretation regarding the material characterization. All authors reviewed the results and approved the final version of the manuscript.

## Conflict of Interest

The authors declare that the research was conducted in the absence of any commercial or financial relationships that could be construed as a potential conflict of interest.

## Supplementary Material

FIGURE S1 Number of Ki67^+^ cells/spheroid. The number of Ki67^+^ cells was manually counted on stained histological sections (5 μm) of the spheroids. Data are represented as mean ± 95% CI, significant differences (*p* < 0.05) were marked ^∗^ (*n* = 6, Kruskal–Wallis test).

## References

[B1] Achilli T.-M. Meyer J. Morgan J. R. (2012). Advances in the formation, use and understanding of multi-cellular spheroids. *Expert Opin. Biol. Ther.* 12 1347–1360. 10.1517/14712598.2012.707181 22784238 PMC4295205

[B2] Alvarez-Pérez J. Ballesteros P. Cerdán S. (2005). Microscopic images of intraspheroidal pH by 1H magnetic resonance chemical shift imaging of pH sensitive indicators. *Magn. Reson. Mater. Phys. Biol. Med.* 18 293–301. 10.1007/s10334-005-0013-z 16328228

[B3] Anderer U. Libera J. (2002). In vitro engineering of human autogenous cartilage. *J. Bone Miner. Res.* 17 1420–1429. 10.1359/jbmr.2002.17.8.1420 12162496

[B4] Armoiry X. Cummins E. Connock M. Metcalfe A. Royle P. Johnston R. (2018). Autologous chondrocyte Implantation with chondrosphere for treating articular cartilage defects in the knee: an evidence review group perspective of a NICE single technology appraisal. *Pharmacoeconomics* 37 879–886. 10.1007/s40273-018-0737-z 30426462

[B5] Bartz C. Meixner M. Giesemann P. Roël G. Bulwin G.-C. Smink J. J. (2016). An ex vivo human cartilage repair model to evaluate the potency of a cartilage cell transplant. *J. Transl. Med.* 14:317 10.1186/s12967-016-1065-8 PMC5111252 27846904

[B6] Basad E. Wissing F. R. Fehrenbach P. Rickert M. Steinmeyer J. Ishaque B. (2015). Matrix-induced autologous chondrocyte implantation (MACI) in the knee: clinical outcomes and challenges. *Knee Surg. Sport Traumatol. Arthrosc.* 23 3729–3735. 10.1007/s00167-014-3295-3298 25218576

[B7] Becher C. Laute V. Fickert S. Zinser W. Niemeyer P. John T. (2017). Safety of three different product doses in autologous chondrocyte implantation: results of a prospective, randomised, controlled trial. *J. Orthop. Surg. Res.* 12:71 10.1186/s13018-017-0570-577 PMC5429514 28499391

[B8] Berneel E. Philips C. Declercq H. Cornelissen R. (2016). Redifferentiation of high-throughput generated fibrochondrocyte micro-aggregates: impact of low oxygen tension. *Cells. Tissues Organs* 202 369–381. 10.1159/000447509 27536780

[B9] Billiet T. Gevaert E. De Schryver T. Cornelissen M. Dubruel P. (2014). The 3D printing of gelatin methacrylamide cell-laden tissue-engineered constructs with high cell viability. *Biomaterials* 35 49–62. 10.1016/j.biomaterials.2013.09.078 24112804

[B10] Caron M. M. J. Emans P. J. Coolsen M. M. E. Voss L. Surtel D. A. M. Cremers A. (2012). Redifferentiation of dedifferentiated human articular chondrocytes: comparison of 2D and 3D cultures. *Osteoarthr. Cartil.* 20 1170–1178. 10.1016/j.joca.2012.06.016 22796508

[B11] Cigan A. D. Roach B. L. Nims R. J. Tan A. R. Albro M. B. Stoker A. M. (2016). High seeding density of human chondrocytes in agarose produces tissue-engineered cartilage approaching native mechanical and biochemical properties. *J. Biomech.* 49 1909–1917. 10.1016/j.jbiomech.2016.04.039 27198889 PMC4920373

[B12] Daly A. C. Critchley S. E. Rencsok E. M. Kelly D. J. Sophia Fox A. J. (2016). A comparison of different bioinks for 3D bioprinting of fibrocartilage and hyaline cartilage. *Biofabrication* 8:045002 10.1088/1758-5090/8/4/045002 27716628

[B13] Davies R. Kuiper N. (2019). Regenerative medicine: a review of the evolution of autologous chondrocyte implantation (ACI) therapy. *Bioengineering* 6:22 10.3390/bioengineering6010022 PMC6466051 30871236

[B14] De Moor L. Beyls E. Declercq H. (2019). Scaffold free microtissue formation for enhanced cartilage repair. *Ann. Biomed. Eng.* 48 298–311. 10.1007/s10439-019-02348-4 31451988

[B15] De Moor L. Merovci I. Baetens S. Verstraeten J. Kowalska P. Krysko D. V. (2018). High-throughput fabrication of vascularized spheroids for bioprinting. *Biofabrication* 10:035009 10.1088/1758-5090/aac7e6 29798932

[B16] Deng Z. Jin J. Zhao J. Xu H. (2016). Cartilage defect treatments: with or without cells? mesenchymal stem cells or chondrocytes? Traditional or matrix-assisted? A systematic review and meta-analyses. *Stem Cells Int.* 2016:9201492 10.1155/2016/9201492 PMC4709777 26839570

[B17] Dewan A. K. Gibson M. A. Elisseeff J. H. Trice M. E. (2014). Evolution of autologous chondrocyte repair and comparison to other cartilage repair techniques. *Biomed Res. Int.* 2014:272481 10.1155/2014/272481 PMC4151850 25210707

[B18] Ewa-Choy Y. W. Pingguan-Murphy B. Abdul-Ghani N. A. Jahendran J. Chua K. H. (2017). Effect of alginate concentration on chondrogenesis of co-cultured human adipose-derived stem cells and nasal chondrocytes: a biological study. *Biomater. Res.* 21:19 10.1186/s40824-017-0105-107 PMC5646124 29075508

[B19] Farag M. M. (2017). “Design and manufacture of biodegradable products from renewable resources,” in *Handbook of Composites From Renewable Materials*, eds Kumar V. Thakur M. K. Kessler M. R. (Hoboken, NJ: John Wiley & Sons, Inc), 111–131. 10.1002/9781119441632.ch23

[B20] Foldager C. B. Gomoll A. H. Lind M. Spector M. (2012). Cell seeding densities in autologous chondrocyte implantation techniques for cartilage repair. *Cartilage* 3 108–117. 10.1177/1947603511435522 26069624 PMC4297130

[B21] Fox A. J. S. Bedi A. Rodeo S. A. (2009). The basic science of articular cartilage: structure, composition, and function. *Sports Health* 1 461–468. 10.1177/1941738109350438 23015907 PMC3445147

[B22] Fox A. J. S. Bedi A. Rodeo S. A. (2012). The basic science of human knee menisci: structure, composition, and function. *Sports Health* 4 340–351. 10.1177/1941738111429419 23016106 PMC3435920

[B23] Gaskell H. Sharma P. Colley H. E. Murdoch C. Williams D. P. Webb S. D. (2016). Characterization of a functional C3A liver spheroid model. *Toxicol. Res.* 5 1053–1065. 10.1039/c6tx00101g PMC5047049 27746894

[B24] Gevaert E. Dollé L. Billiet T. Dubruel P. van Grunsven L. van Apeldoorn A. (2014). High throughput micro-well generation of hepatocyte micro-aggregates for tissue engineering. *PLoS One* 9:e105171 10.1371/journal.pone.0105171 PMC4136852 25133500

[B25] Ghosh S. Laha M. Mondal S. Sengupta S. Kaplan D. L. (2009). In vitro model of mesenchymal condensation during chondrogenic development. *Biomaterials* 30 6530–6540. 10.1016/j.biomaterials.2009.08.019 19732950 PMC2760153

[B26] Gionet-Gonzales M. A. Leach J. K. (2018). Engineering principles for guiding spheroid function in the regeneration of bone, cartilage, and skin. *Biomed. Mater.* 13:034109 10.1088/1748-605X/aab0b3 PMC5898817 29460842

[B27] Grogan S. P. Dorthé E. W. Glembotski N. E. Gaul F. D’Lima D. D. (2019). Cartilage tissue engineering combining microspheroid building blocks and microneedle arrays. *Connect. Tissue Res.* 61 229–243. 10.1080/03008207.2019.1617280 31134817

[B28] Hu X. Ma L. Wang C. Gao C. (2009). Gelatin hydrogel prepared by photo-initiated polymerization and loaded with TGF-beta1 for cartilage tissue engineering. *Macromol. Biosci.* 9 1194–1201. 10.1002/mabi.200900275 19890886

[B29] Huang B. J. Hu J. C. Athanasiou K. A. (2016). Cell-based tissue engineering strategies used in the clinical repair of articular cartilage. *Biomaterials* 98 1–22. 10.1016/j.biomaterials.2016.04.018 27177218 PMC4899115

[B30] Jo C. H. Lee Y. G. Shin W. H. Kim H. Chai J. W. Jeong E. C. (2014). Intra-articular injection of mesenchymal stem cells for the treatment of osteoarthritis of the knee: a proof-of-concept clinical trial. *Stem Cells* 32 1254–1266. 10.1002/stem.1634 24449146

[B31] Knutsen G. Drogset J. O. Engebretsen L. Grøntvedt T. Isaksen V. Ludvigsen T. C. (2007). A randomized trial comparing autologous chondrocyte implantation with microfracture. Findings at five years. *J. Bone Joint Surg. Am.* 89 2105–2112. 10.2106/JBJS.G.00003 17908884

[B32] Krill M. Early N. Everhart J. S. Flanigan D. C. (2018). Autologous chondrocyte implantation (ACI) for knee cartilage defects. *JBJS Rev.* 6:e5 10.2106/JBJS.RVW.17.00078 29461987

[B33] Levato R. Webb W. R. Otto I. A. Mensinga A. Zhang Y. van Rijen M. (2017). The bio in the ink: cartilage regeneration with bioprintable hydrogels and articular cartilage-derived progenitor cells. *Acta Biomater.* 61 41–53. 10.1016/J.ACTBIO.2017.08.005 28782725 PMC7116023

[B34] Li J. Chen G. Xu X. Abdou P. Jiang Q. Shi D. (2019). Advances of injectable hydrogel-based scaffolds for cartilage regeneration. *Regen. Biomater.* 6 129–140. 10.1093/rb/rbz022 31198581 PMC6547311

[B35] Liu J. Hilderink J. Groothuis T. A. M. Otto C. van Blitterswijk C. A. de Boer J. (2015). Monitoring nutrient transport in tissue-engineered grafts. *J. Tissue Eng. Regen. Med.* 9 952–960. 10.1002/term.1654 23349072

[B36] Loessner D. Meinert C. Kaemmerer E. Martine L. C. Yue K. Levett P. A. (2016). Functionalization, preparation and use of cell-laden gelatin methacryloyl-based hydrogels as modular tissue culture platforms. *Nat. Protoc.* 11 727–746. 10.1038/nprot.2016.037 26985572

[B37] Markovic M. Van Hoorick J. Hölzl K. Tromayer M. Gruber P. Nürnberger S. (2015). Hybrid tissue engineering scaffolds by combination of three-dimensional printing and cell photoencapsulation. *J. Nanotechnol. Eng. Med.* 6 0210011–0210017. 10.1115/1.4031466 26858826 PMC4714880

[B38] Mironov V. Visconti R. P. Kasyanov V. Forgacs G. Drake C. J. Markwald R. R. (2009). Organ printing: tissue spheroids as building blocks. *Biomaterials* 30 2164–2174. 10.1016/j.biomaterials.2008.12.084 19176247 PMC3773699

[B39] Moldovan N. Maldovan L. Raghunath M. (2018). Of balls, inks and cages: hybrid biofabrication of 3D tissue analogs. *Int. J. Bioprint.* 5:1 10.18063/ijb.v5i1.167 PMC7294696 32596531

[B40] Moldovan N. I. Hibino N. Nakayama K. (2017). Principles of the kenzan method for robotic cell spheroid-based three-dimensional bioprinting. *Tissue Eng. Part B Rev.* 23 237–244. 10.1089/ten.teb.2016.0322 27917703

[B41] Mouser V. H. M. Levato R. Mensinga A. Dhert W. J. A. Gawlitta D. Malda J. (2018). Bio-ink development for three-dimensional bioprinting of hetero-cellular cartilage constructs. *Connect. Tissue Res.* 61 137–151. 10.1080/03008207.2018.1553960 30526130

[B42] Nichol J. W. Khademhosseini A. (2009). Modular tissue engineering: engineering biological tissues from the bottom up. *Soft Matter.* 5 1312–1319. 10.1039/b814285h 20179781 PMC2826124

[B43] Onofrillo C. Duchi S. O’Connell C. D. Blanchard R. O’Connor A. J. Scott M. (2018). Biofabrication of human articular cartilage: a path towards the development of a clinical treatment. *Biofabrication* 10:045006 10.1088/1758-5090/aad8d9 30088479

[B44] Pahoff S. Meinert C. Bas O. Nguyen L. Klein T. J. Hutmacher D. W. (2019). Effect of gelatin source and photoinitiator type on chondrocyte redifferentiation in gelatin methacryloyl-based tissue-engineered cartilage constructs. *J. Mater. Chem. B.* 7:2607 10.1039/C8TB02607F 32254918

[B45] Parfenov V. A. Koudan E. V. Bulanova E. A. Karalkin P. A. Das Pereira F. Norkin N. E. (2018). Scaffold-free, label-free and nozzle-free biofabrication technology using magnetic levitational assembly. *Biofabrication* 10:034104 10.1088/1758-5090/aac900 29848793

[B46] Pepelanova I. Kruppa K. Scheper T. Lavrentieva A. (2018). Gelatin-methacryloyl (GelMA) hydrogels with defined degree of functionalization as a versatile toolkit for 3D cell culture and extrusion bioprinting. *Bioengineering* 5:55 10.3390/bioengineering5030055 PMC6165498 30022000

[B47] Radisic M. Malda J. Epping E. Geng W. Langer R. Vunjak-Novakovic G. (2006). Oxygen gradients correlate with cell density and cell viability in engineered cardiac tissue. *Biotechnol. Bioeng.* 93 332–343. 10.1002/bit.20722 16270298

[B48] Roosens A. Handoyo Y. P. Dubruel P. Declercq H. (2019). Impact of modified gelatin on valvular microtissues. *J. Tissue Eng. Regen. Med.* 13 771–784. 10.1002/term.2825 30770648

[B49] Roosens A. Puype I. Cornelissen R. (2017). Scaffold-free high throughput generation of quiescent valvular microtissues. *J. Mol. Cell. Cardiol.* 106 45–54. 10.1016/j.yjmcc.2017.03.004 28322869

[B50] Rothrauff B. B. Shimomura K. Gottardi R. Alexander P. G. Tuan R. S. (2017). Anatomical region-dependent enhancement of 3-dimensional chondrogenic differentiation of human mesenchymal stem cells by soluble meniscus extracellular matrix. *Acta Biomater.* 49 140–151. 10.1016/j.actbio.2016.11.046 27876676 PMC5543932

[B51] Rouillard A. D. Berglund C. M. Lee J. Y. Polacheck W. J. Tsui Y. Bonassar L. J. (2011). Methods for photocrosslinking alginate hydrogel scaffolds with high cell viability. *Tissue Eng. Part C Methods* 17 173–179. 10.1089/ten.tec.2009.0582 20704471

[B52] Salamon A. van Vlierberghe S. van Nieuwenhove I. Baudisch F. Graulus G.-J. Benecke V. (2014). Gelatin-based hydrogels promote chondrogenic differentiation of human adipose tissue-derived mesenchymal stem cells in vitro. *Materials* 7 1342–1359. 10.3390/ma7021342 28788517 PMC5453082

[B53] Schon B. S. Hooper G. J. Woodfield T. B. F. (2017). Modular tissue assembly strategies for biofabrication of engineered cartilage. *Ann. Biomed. Eng.* 45 100–114. 10.1007/s10439-016-1609-3 27073109

[B54] Schuurman W. Levett P. A. Pot M. W. van Weeren P. R. Dhert W. J. A. Hutmacher D. W. (2013). Gelatin-methacrylamide hydrogels as potential biomaterials for fabrication of tissue-engineered cartilage constructs. *Macromol. Biosci.* 13 551–561. 10.1002/mabi.201200471 23420700

[B55] Shin Y.-S. Yoon J.-R. Kim H.-S. Lee S.-H. (2018). Intra-articular injection of bone marrow-derived mesenchymal stem cells leading to better clinical outcomes without difference in mri outcomes from baseline in patients with knee osteoarthritis. *Knee Surg. Relat. Res.* 30 206–214. 10.5792/ksrr.17.201 29983008 PMC6122947

[B56] Susienka M. J. Wilks B. T. Morgan J. R. (2016). Quantifying the kinetics and morphological changes of the fusion of spheroid building blocks. *Biofabrication* 8:045003 10.1088/1758-5090/8/4/045003 27721222

[B57] Tan G.-K. Dinnes D. L. M. Myers P. T. Cooper-White J. J. (2011). Effects of biomimetic surfaces and oxygen tension on redifferentiation of passaged human fibrochondrocytes in 2D and 3D cultures. *Biomaterials* 32 5600–5614. 10.1016/j.biomaterials.2011.04.033 21592565

[B58] Tytgat L. Baudis S. Ottevaere H. Liska R. Thienpont H. Dubruel P. (2018). “Photopolymerizable materials for cell encapsulation,” in *3D Printing and Biofabrication*, eds Ovsianikov A. Yoo J. Mironov V. (Berlin: Springer International Publishing), 353–396. 10.1007/978-3-319-45444-3_15

[B59] Tytgat L. Van Damme L. Hoorick J. Van, Declercq H. Thienpont H. (2019). Additive manufacturing of photo-crosslinked gelatin scaffolds for adipose tissue engineering. *Acta Biomater.* 94 340–350. 10.1016/j.actbio.2019.05.062 31136829

[B60] Ude C. C. Chen H. C. Norhamdan M. Y. Azizi B. M. Aminuddin B. S. Ruszymah B. H. I. (2017). The evaluation of cartilage differentiations using transforming growth factor beta3 alone and with combination of bone morphogenetic protein-6 on adult stem cells. *Cell Tissue Bank* 18 355–367. 10.1007/s10561-017-9638-9631 28667462

[B61] Van Den Bulcke A. I. Bogdanov B. De Rooze N. Schacht E. H. Cornelissen M. Berghmans H. (2000). Structural and rheological properties of methacrylamide modified gelatin hydrogels. *Biomacromolecules* 1 31–38. 10.1021/bm990017d 11709840

[B62] Van Hoorick J. Gruber P. Markovic M. Tromayer M. Van Erps J. Thienpont H. (2017). Cross-linkable gelatins with superior mechanical properties through carboxylic acid modification: increasing the two-photon polymerization potential. *Biomacromolecules* 18 3260–3272. 10.1021/acs.biomac.7b00905 28850786 PMC5647566

[B63] Vega S. L. Kwon M. Y. Burdick J. A. (2017). Recent advances in hydrogels for cartilage tissue engineering. *Eur. Cells Mater.* 33 59–75. 10.22203/eCM.v033a05 PMC5748291 28138955

[B64] Wang W. Rigueur D. Lyons K. M. (2014). TGFβ signaling in cartilage development and maintenance. *Birth Defect. Res. Part C Embryo Today Rev.* 102 37–51. 10.1002/bdrc.21058 PMC4267887 24677722

[B65] Wintermantel E. Mayer J. Goehring T. N. (2001). Composites for biomedical applications. *Encyclop. Mater. Sci. Technol.* 1 1371–1376. 10.1016/b0-08-043152-6/00255-252

[B66] Woodruff M. A. Hutmacher D. W. (2010). The return of a forgotten polymer—Polycaprolactone in the 21st century. *Prog. Polym. Sci.* 35 1217–1256. 10.1016/j.progpolymsci.2010.04.002

[B67] Žigon-Branc S. Markovic M. Van Hoorick J. Van Vlierberghe S. Dubruel P. Zerobin E. (2019). Impact of hydrogel stiffness on differentiation of human adipose-derived stem cell microspheroids. *Tissue Eng. Part A* 25 1369–1380. 10.1089/ten.TEA.2018.0237 30632465 PMC6784494

