# Supplementary figures and images for: Hybrid Bioprinting of Chondrogenically Induced Human Mesenchymal Stem Cell Spheroids
Source: Front Bioeng Biotechnol. 2020 May 25;8:484. doi: 10.3389/fbioe.2020.00484 (PMC7261943; doi:10.3389/fbioe.2020.00484)

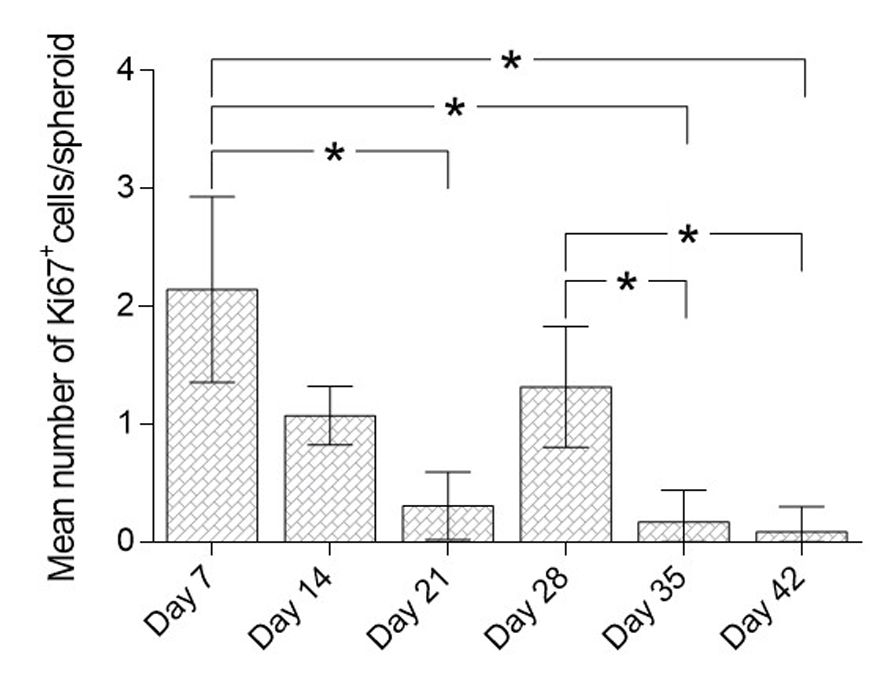

Supplement: FIGURE S1 — Number of Ki67+ cells/spheroid. The number of Ki67+ cells was manually counted on stained histological sections (5 μm) of the spheroids. Data are represented as mean ± 95% CI, significant differences (p < 0.05) were marked ∗ (n = 6, Kruskal–Wallis test). [file Image_1.tif]
